# Hugh Ley

**Published:** 1837-04

**Authors:** 


HUGH LEY, M.D.

On the 24th January, Hugh Ley, M.D. Lecturer on Midwifery at St. Bartholomew's Hospital, in the forty-seventh year of his age. Dr. Ley was the son of a physician and was born at Abingdon, in Berkshire, in the year 1790. He studied first in London, and then at Edinburgh, where he graduated in 1813, his thesis being "De natura intima phthiseos pulmonalis." Shortly after settling in London, he was elected physician to the Westminster Lying-in Hospital. Subsequently to this he became associated with Dr. Merriman, in giving lectures on midwifery and the diseases of women and children at the Middlesex Hospital, and was appointed assistant physician accoucheur to that institution; and, on Dr. Merriman's resignation, he was unanimously elected physician in his stead. He was appointed to the obstetric chair at St. Bartholomew's in the autumn of 1835. He died of an affection of the heart, consequent on acute rheumatism.

Dr. Ley was the author of several valuable papers in the Medical Gazette. His only separate publication is the "Essay on Laryngismus Stridulus," of which we gave a very full account in the third number of this journal.

Dr. Ley's professional character (as is truly stated by Mr. Earle,) was deservedly high, and without blemish; his conduct and sentiments on all subjects were those of a gentleman. [*Abridged from Mr. Earle's Speech at the Medico-Chirurgical Society on resigning the Presidency, February 28th*, 1837.]

